# From coding to conclusions: lessons for researchers using routinely collected primary care data

**DOI:** 10.3399/BJGP.2025.0560

**Published:** 2026-05-01

**Authors:** Sharon Dixon, Anna E Seeley

**Affiliations:** 1 Nuffield Department of Primary Care Health Sciences, University of Oxford, Oxford, UK

## Introduction

Medical research increasingly uses routinely collected electronic health records (EHRs), a seemingly efficient approach to accessing real world data. Within the UK, large data repositories such as QResearch, Clinical Practice Research Datalink (CPRD), and OpenSAFELY, pool primary care records from millions of patients for use in research. Primary care EHR data can include codes created within a variety of coding systems such as SNOMED. These record medications prescribed, problems, symptoms, diagnoses (confirmed or suspected), consultation frequency, investigations, and other clinical observations (for example, height and weight), as well as demographic and personal variables (such as ethnicity, smoking status, and alcohol use). These data are used for multiple purposes, for example, to examine patterns and trends of disease or healthcare access,^1^ assess medicine and vaccine safety,^2^ or build prediction models to identify those at risk of future illness.^3^ Increasingly, these data are being used to simulate randomised controlled trials, and so to examine causality.^4^

The codes found in EHRs are highly dependent on coding practices. All primary care clinicians recognise that what is coded in the patient record is often just a fragment of what happens inside the consultation room. Previous commentaries have focused on how primary care can improve the volume and accuracy of coding^5^ for both their own (visible, known) use of data and the onward (invisible, external) purposes in research. Increasingly, artificial intelligence (AI) is also seen as a solution, removing the ‘human’ in data recording and enabling automated coding. Good coding is part of good care. It allows the mutually agreed framing of clinical presentations and is a vital part of communication across and between care organisations. However, a focus on increasing the proportion of coded data risks overlooking how the application of a clinical code is neither neutral nor inert, but actively reflects the inherent uncertainty in modern day general practice.

In this article we reflect, as both epidemiologists and clinically active GPs, on how codes are used in medical research and consider when relying on coded data may be problematic, using symptom coding as an exemplar. We discuss whether current research practice is doing enough to recognise the nuance of coding in interpretation of findings, and reflect on potential solutions.

## How do researchers use coded primary care EHR data?

Epidemiologists bring together groups of codes from the GP record (‘code lists’) to generate discreet clinical entities or variables that are then used to identify and represent patients with certain characteristics. For example, ‘diabetic patients’ would all have at least one of a selected list of diabetes codes (for example, ‘type 2 diabetes mellitus’, but could include ‘overdue diabetic medication review’ or ‘did not attend diabetic eye screening’) in their notes. Understanding which codes have been used in a research study is key to research reproducibility and transparency. The 2015 RECORD reporting guidelines state that researchers should publish code lists describing how their study population was identified.^6^ We audited all articles published in 2024 using CPRD data (chosen because it is the most established and used primary care data repository). Strikingly, only 51% (*n* = 60/117) of research articles had published code lists, meaning that for half of those published articles it was impossible to ascertain on what basis the research had been conducted.

## The use of symptom codes in EHR research

As Jutel writes, *‘Diagnoses are the classification tools of medicine* [...] *Being diagnosed gives permission to be ill.’* ^7^ Making a diagnosis sets the clinician apart from a lay person or patient, and structures onwards care, together with enabling access to service and status. The diagnostic process is layered, complex, and evolves through time. Diagnostic processes deploy a range of classification tools including deviation from statistical or physiological norms and presence or absence of physical structures or constellations of symptoms. In contrast, coding is binary — it is either present or not. To mitigate this, clinicians commonly code symptoms in place of a diagnosis, or simply refrain from coding at all. The use of symptom coding in EHR research therefore requires particular consideration of the context in which codes arise.

## How, when, and why do clinicians code symptoms?

Symptom coding is a complex, nuanced, and unpredictable process. While some codes in GP records are relatively dichotomous (for example, history of myocardial infarction), others are more heterogeneous, both in terms of use and interpretation. Symptoms of ill health, for example, ‘fatigue’, are an exemplar of this. In general practice, where we see patients with symptoms across multiple possible conditions, descriptive coding can be a deliberate strategy to account for multiple possible outcomes and minimise the risk of diagnostic overshadowing.^8^ Symptom codes can be actively selected as a means of holding uncertainty and to communicate this to subsequent clinicians or viewers of the record. This is vital in a context of eroding relational continuity of care and a growing necessity to nurture informational continuity.^9^ Clinicians may preferentially code some symptoms, giving prominence to ‘red flags’, when they suspect the underlying diagnosis, and chose not to code where they do not.^10^ Finally, the use of symptom codes may be deployed to avoid the consequences of labelling a condition. For example, this can be relevant to mental health conditions, where overuse of diagnostic labels can have implications for individual patients (for example, use in occupational health and insurance forms) together with additional work for GPs under Quality and Outcomes Framework rules.^11^ These nuances of coding are overlain by safety and privacy considerations in data entry, influencing, for example, codes entered in relation to safeguarding, mental health, or contraception.

## How are symptom codes used in EHR research?

In our audit of 2024 CPRD articles, approximately one-third of articles that provided code lists used symptom codes within their research. While some clearly described what they were studying as symptoms, for example, in mapping patterns of symptoms and care presentations before diagnoses of cancer,^12^ others included symptom codes buried within code lists for medical diagnoses. Examples of the latter include ‘H/O bronchitis’, ‘chest cold’, and ‘respiratory symptoms’ within a (diagnostic) variable for ‘lower respiratory tract infection’ (albeit alongside an antibiotic prescription),^13^ or ‘complains of feeling unhappy’ within a code for (diagnosed) depression.^14^ While there may be reasons for choosing broad inclusion criterion, for example, ensuring data inclusion completeness, these are not typically discussed. When the undifferentiated symptom and the diagnostic label are conflated within a single epidemiological code list they are effectively deemed synonymous with each other, which risks minimising nuance and opportunities to study different coding patterns.

## Wider determinants of coding in primary care records

A strength of using primary care EHRs in research is that the overall population represented in these datasets is broadly representative of the UK population. However, these data only reflect those who access care. There is a well-documented inverse care law in general practice, which affects access to GP appointments and therefore impacts on representation within coded data. In addition, GP coding reflects wider social and structural determinants of health and is subject to a number of biases. Doctors are not inert but also live in the world in which they work and are also exposed to the societal influences. Social acceptability, stigma, and recall bias may all affect what is said, what is heard, and what is recorded in the notes. What is safe to add to records, which may now be freely viewed online, adds another level of complexity and potential source of bias.^15^ Social and cultural norms, embedded discrimination, and media trends also influence use of primary care outside the consultation room. These features, linked to individuals, a particular time, place, or local community, becomes homogenised within datasets that represent millions of people over many years.

Variables such as ethnicity are often incomplete or missing and while researchers have developed statistical modelling techniques to get around missing data, these are typically predicated on an assumption that the data are missing at random, rather than shaped by social determinants.

Box 1 summarises the problems outlined above using a specific example from our research.^16^

**Box 1.** An example of the problems of using symptom codes for EHR research

**Patient perspective and epidemiological research question**	**Problems with symptom coding**
**Patient perspective: ‘Could my period pain be endometriosis?’** Sam is 14 years old. Their periods are increasingly painful and are impacting on them at school. They haven’t found over-the-counter analgesics helpful. Their periods are also very heavy. Sam feels very tired and their mum wonders if they might be anaemic. Sam has Googled period pain and has read about endometriosis and wants to know if this might be the cause. **Epidemiological research question**: What evidence is there to help answer Sam’s question? Or to help the clinician know how to respond? What is the pattern of menstrual symptoms in adolescence? And which of these might be associated with the likelihood of a subsequent diagnosis of endometriosis? This study will use QResearch to explore these patterns and relationships. Heavy bleeding, irregular bleeding, and painful menstrual bleeding can overlap. Might this impact on whether this study could be used to build a tool, based on coded data, to identify those at risk of endometriosis or inform decisions about referral for tests and further care?^21^	**Hierarchy of coding** Typically only one symptom would be coded when they are seen as aligned within one presentation, and the drivers can be complex and contextual. Would you code anaemia? Tiredness? Heavy bleeding? Or dysmenorrhoea? We don’t know how this is chosen. However, if in your locality a pelvic ultrasound scan cannot be requested on the basis of heavy bleeding alone, this becomes a driver towards coding dysmenorrhoea when a scan is indicated (or requested). **Menstrual stigma** There is normalisation and stigma associated with menstruation and menstrual symptoms. Talking about periods is encompassed within a well-documented menstrual etiquette of concealment and coping. The acceptability of talking about periods and about hormonal contraception may vary between individuals. Symptom recording may depend on who comes with Sam to the appointment, which clinician sees them, and who has access to their online record **Absence of coded symptoms does not equal absence of disease or of symptoms (or mean a GP was not consulted about them)** **Population prevalence versus coded frequencies** Community surveys put the prevalence of adolescent dysmenorrhoea at about 70%–94%, with up to a third experiencing severe symptoms, for example, causing school absence.^16^ In this study, we see that <4% of teenagers have dysmenorrhoea coded, with disparities by ethnicity and Townsend Quintile of deprivation. There is no evidence that this represents a difference in prevalence, and is likely complicated by access to or perceived accessibility of care.^16^ **Endometriosis is not diagnosed equally** Endometriosis diagnoses in adult women in NHS hospitals show a U-shaped curve, likely reflecting less access to care in the lowest quintile of deprivation (most deprived), and the use of private services in the highest (least deprived).^22^ This is relevant in a context of long waiting lists for gynaecology and sparse adolescent gynaecology services.

EHR = electronic health record. NICE = National Institute for Health and Care Excellence.

## Moving forward

We advocate that all research articles using EHR data, and particularly those that include symptom codes, should discuss the potential limitations of codes chosen. This requires publishing code lists so that they are easily accessible, and for this to be a shared responsibility between researchers, reviewers, and publishers. This is in accord with RECORD guidelines and would facilitate transparent dialogue about equity and inclusion in research.

This could be supported by greater involvement of primary care clinicians in the research process. In our audit only a minority of studies were co-authored by a UK GP or primary care practitioner. Research specialists, secondary care clinicians, and international authors may lack the working knowledge of how codes come into being, and so fail to appreciate the nuances of how the data they are using has been created. Examples we identified in our audit that speak to a lack of familiarity with UK GP records and NHS primary care include presuming glucosamine would be typically and preferentially prescribed rather than purchased,^17^ and the conjecture that all members of a household will be co-registered at one practice and easily identifiable.^18^ These would likely be recognised by NHS GPs as potentially flawed assumptions.

## Will AI help?

Using automated tools for data collection and curation is a proposed solution, not least because of the high workloads in general practice. AI scribes such as Heidi (which audiorecords and transcribes consultation talk) are already available in UK primary care. Evidence suggests they both increase the amount of information recorded and coded in any given consultation.^19^ While these tools may reduce preferential coding and facilitate better documentation, removing clinician oversight may lead to unintended consequences.^20^ The rapid expansion of recorded information could create problems for both clinicians and researchers to sift through. It also requires that any prompts used for automated coding incorporate nuance. For instance, how often tiredness is mentioned in a consultation versus under what circumstances the code ‘fatigue’ might be used as a unifying problem code may differ.

While automation could provide overall ‘narrative coherence’ by applying unifying codes, this might compromise GPs’ ability to hold uncertainty, for example, wishing to delay coding a diagnosis until this is deemed accurate or communicated carefully to the patient.^20^ It is uncertain how AI will navigate the diversity of authors (clinicians and patients) who contribute to the medical record, or what this will mean for coding and epidemiological research. The deployment of online communication tools, such as e-consultation and AccuRx, allows patients to write their own accounts of symptoms and diagnostic hypotheses or concerns, and these are entered automatically into the notes where they may be coded and enter research datasets. It is not clear what impacts these will have on the evolving EHR research database, nor how and whether AI will learn to differentiate between patient and practitioner data entries.

## Conclusion

We welcome the significant and growing use of EHR data and recognise it’s potential in healthcare research. However, achieving this potential also requires recognising and mitigating against data limitations. We have used symptom coding and our audit of research using CPRD to illustrate how decisions about coding are complex, situational, and sometimes problematic. Symptom coding is highly variable, and both selection and active deployment likely differs from recording medical diagnoses. Conflating these without visibility within diagnostic code groups might matter. We have shown how research using EHR data can fail to recognise or pay attention to this, which risks creating new, or entrenching existing, inequalities. While we acknowledge that efforts to improve data curation in primary care are important, we argue that EHR researchers also need to take care in how they use clinical codes, with greater transparency in methods, including code list derivation, and reporting in their research.
